# Subgaleal Lipoma: Imaging Findings

**DOI:** 10.5334/jbsr.2372

**Published:** 2021-04-20

**Authors:** Mihaela-Magdalena Vlad, Michaël Dupont, Françoise Kayser

**Affiliations:** 1 Université catholique de Louvain, CHU UCL Namur, Department of Radiology, 1 Avenue Dr G Thérasse, 5530, Yvoir, BE

**Keywords:** subgaleal lipoma, galea aponeurosis, epicranial aponeurosis, scalp mass, internal echogenic lines

## Abstract

Subgaleal lipoma is a benign tumor of adipose tissue. It should be suspected when a semi-spherical avascular mass with well-defined margins, iso- or hyperechoic in most cases, with thin internal echogenic lines parallel to the long axis of the tumor, is observed between the galea aponeurosis and periosteum of the cranial bone. We report a series of cases of three patients who underwent surgical lesion excision and whose histopathological examination findings confirmed the diagnosis of lipoma.

**Main Teaching Point:**

The presence of long continuous echogenic lines within a lens-shaped soft tissue mass located beneath the galea aponeurosis may suggest the diagnosis of subgaleal lipoma.

## Introduction

Scalp masses are commonly encountered in clinical practice. Ultrasound is usually the first imaging technique used to investigate soft tissue masses of the scalp due to its accessibility and affordability. Lipoma of the scalp can be easily diagnosed on ultrasound because of its semi-spherical shape and the presence of thin internal echogenic lines parallel to the long axis of the tumor [1]. Subgaleal lipoma is recognized by the location between the galea aponeurosis and the cranial bone, most of them being reported in the forehead [2,3].

We report the imaging findings of subgaleal lipomas in three patients, reviewing the imaging characteristics of these lesions.

## Results

Our three patients were aged between 39 and 64 years at the time of diagnosis. The localization, size, and echogenicity of the lesions are presented in ***Table 1***. All patients underwent surgical lesion excision, and histopathological examination confirmed the diagnosis of lipoma. The echogenicity of the lesions, compared to that of the adjacent fat on ultrasound, varied from one patient to the other. However, all lesions showed thin internal echogenic lines, and none showed calcifications or internal Doppler flow.

**Table 1:** Characteristics of the lesions according to ultrasonographic findings.


CHARACTERISTICS	PATIENT 1	PATIENT 2	PATIENT 3

Age (years)	39	63	64

Sex	Male	Female	Male

Localization	Left parietal	Forehead	Occipital

Size	23 × 7 mm	18 × 6 mm	22 × 7 mm

Echogenicity	Hyperechoic	Isoechoic	Hypoechoic

Internal linear echogenic lines	Yes	Yes	Yes

Calcification	None	None	None

Internal Doppler flow	None	None	None

Imaging workup	US, CT	US, MRI	US

Pathological sampling	Yes	Yes	Yes


**Figure 1:**
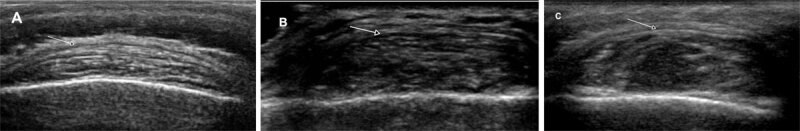
Grayscale ultrasound images showing a solid mass (lens-shaped or semi-spherical) that is hyperechoic in patient 1, **(A)** isoechoic in patient 2, and **(B)** hypoechoic in patient 3, **(C)** with thin longitudinal internal echogenic lines parallel to the long axis of lesion. Galea aponeurosis (white arrow) appears as a fine linear hypoechoic structure between the mass and the subcutaneous fat of the scalp.

**Figure 2:**
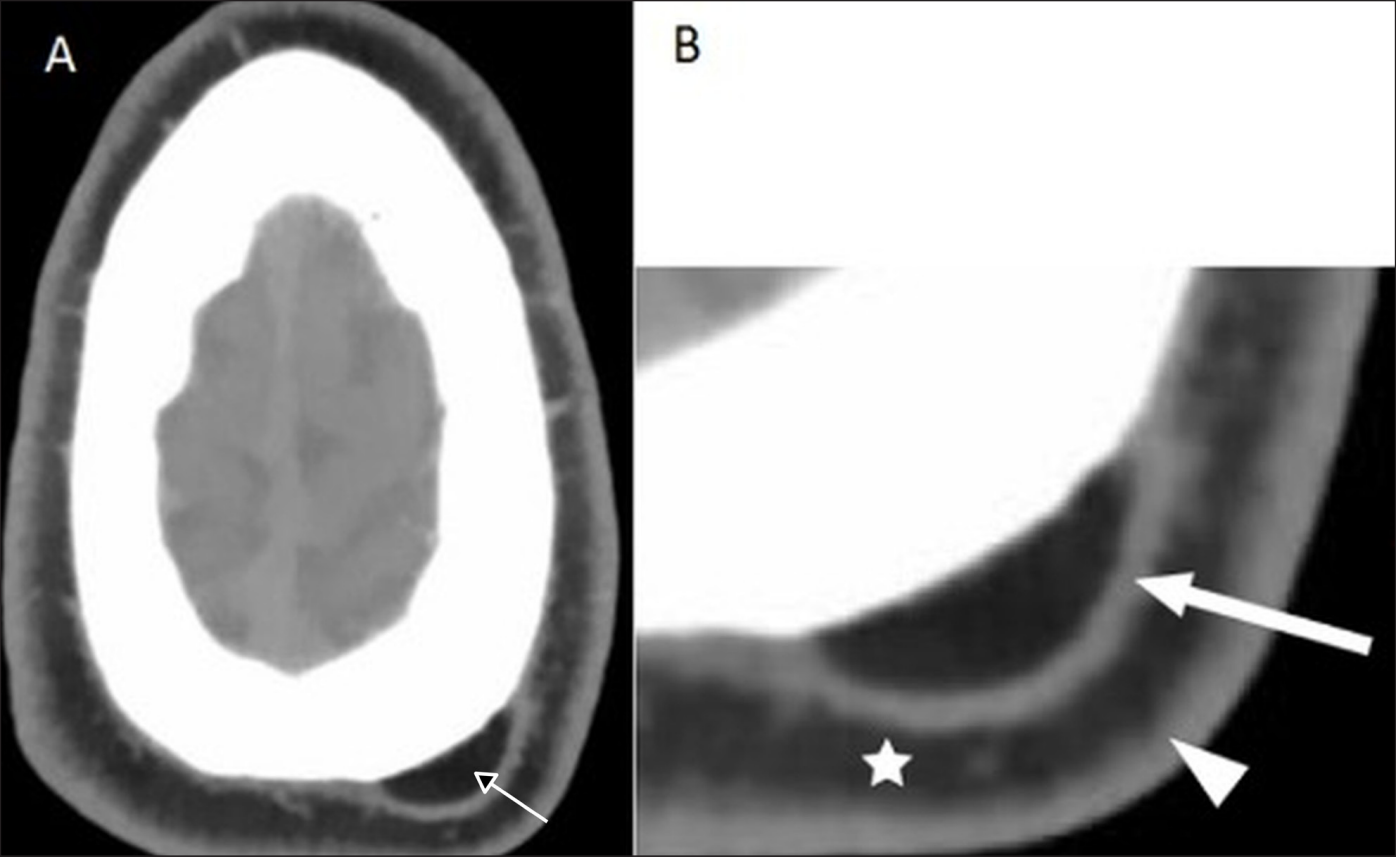
Unenhanced CT scan (axial view). **(A)** A fat-containing mass located under the galea aponeurosis. **(B)** An enlarged image showing the different layers of the scalp distinguishable on CT imaging: skin (white arrowhead), subcutaneous fat (white star), galea aponeurosis (white arrow).

**Figure 3:**
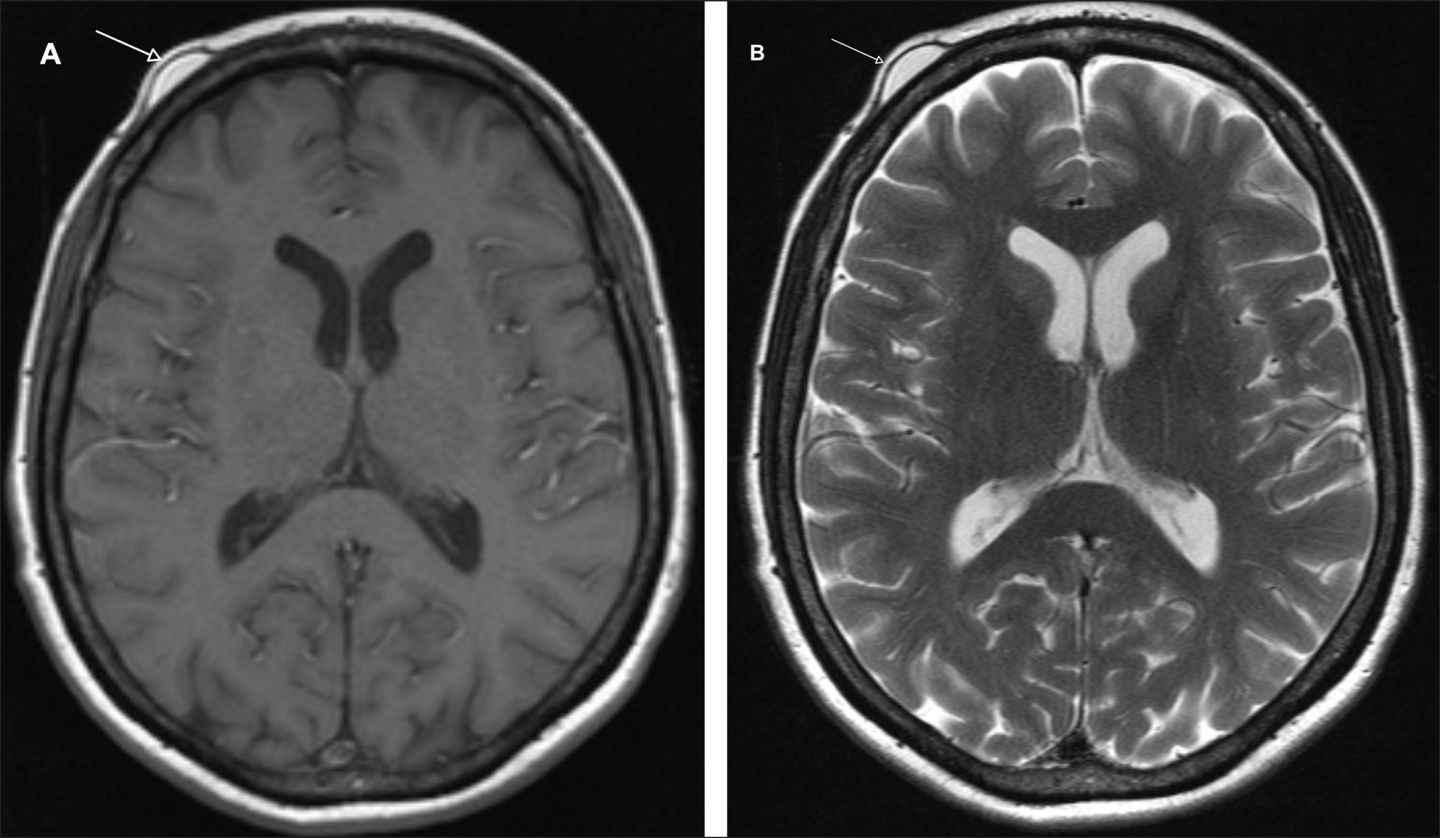
Subgaleal lipoma of the forehead. Axial T1- **(A)** and T2-weighted **(B)** sequences of head MRI showing a lipoma as a hyperintense mass located under the galea aponeurosis (arrow).

The ultrasonographic appearance of these subgaleal lipomas is shown in ***Figure 1***. The computed tomography (CT) appearance of patient 1 is presented in ***Figure 2***. The magnetic resonance imaging (MRI) of patient 2 is shown in ***Figure 3***.

## Discussion

Subgaleal lipoma is a heterotopic tumor of adipose tissue typically occurring between the periosteum and the galea aponeurosis (epicranial aponeurosis) of the scalp [4]. Most of the subgaleal lipomas described in the literature are located in the forehead, but they can also develop in other parts of the scalp as in the case of two patients in our case series [1,4].

Ultrasound is usually the first imaging technique used for soft tissue masses owing to its accessibility and affordability. On ultrasound, the subgaleal lipoma appears as a solid mass, semi-spherical or lens-shaped, with a flat or slightly concave base towards the periosteum of the cranial bone, and a convex surface towards the subcutaneous fat, parallel to the skin surface [1]. Most of these lesions appear iso- or hyperechoic compared to the adjacent fat [1]. They contain multiple thin internal echogenic lines parallel to the long axis of the tumor [1,5].

Besides subgaleal lipoma, the differential diagnosis of scalp lumps also includes epidermoid cysts, dermoid cysts, trichilemmal cysts, intraosseous hemangiomas and other vascular malformations, and malignant lesions such as lymphoma, carcinoma, and metastasis [2].

CT scan is typically used to examine the bone alterations [2]. On unenhanced CT, the lesion appears oval or semi-spherical in shape, well-circumscribed, and homogeneous, presenting a fat attenuation/density of –50 to –100 Hounsfield Units without calcifications [2,5].

MRI can be used to evaluate the extent of the lesion [2]. Subgaleal lipoma shows a high signal on T1- and T2- weighted sequences, with a suppressed signal on fat-saturated sequences.

## Conclusion

On ultrasound, the diagnosis of subgaleal lipoma should be suspected in the presence of a lens-shaped or semi-spherical soft tissue mass located between the galea aponeurosis and periosteum of the cranial bone that is iso- or hyperechoic, with thin continuous echogenic intralesional lines parallel to the long axis of the lesion.
